# PRRG4 regulates mitochondrial function and promotes migratory behaviors of breast cancer cells through the Src-STAT3-POLG axis

**DOI:** 10.1186/s12935-023-03178-0

**Published:** 2023-12-16

**Authors:** Yang Wang, Jieyi Wang, Lan Chen, Zhuo Chen, Tong Wang, Shuting Xiong, Tong Zhou, Guang Wu, Licai He, Jiawei Cao, Min Liu, Hongzhi Li, Haihua Gu

**Affiliations:** 1 Key Laboratory of Laboratory Medicine, Ministry of Education, Wenzhou Key Laboratory of Cancer Pathogenesis and Translation, School of Laboratory Medicine and Life Sciences, Wenzhou Medical University, Wenzhou, 325035 China; 2 Department of Clinical Laboratory, The First Affiliated Hospital of Ningbo University, Ningbo, China; 3 Department of Orthopedics, The Third Affiliated Hospital of Wenzhou Medical University, Wenzhou, 325200 Zhejiang China; 4 School of Laboratory Medicine and Life Sciences, Wenzhou Medical University, Room 903 and 904, Biomedical Research Building-South, Chashan University Town, Wenzhou, 325035 Zhejiang China

**Keywords:** PRRG4, Mitochondrial DNA, Src, STAT3, POLG, Breast cancer

## Abstract

**Background:**

Breast cancer is the leading cause of cancer death for women worldwide. Most of the breast cancer death are due to disease recurrence and metastasis. Increasingly accumulating evidence indicates that mitochondria play key roles in cancer progression and metastasis. Our recent study revealed that transmembrane protein PRRG4 promotes the metastasis of breast cancer. However, it is not clear whether PRRG4 can affect the migration and invasion of breast cancer cells through regulating mitochondria function.

**Methods:**

RNA-seq analyses were performed on breast cancer cells expressing control and PRRG4 shRNAs. Quantitative PCR analysis and measurements of mitochondrial ATP content and oxygen consumption were carried out to explore the roles of PRRG4 in regulating mitochondrial function. Luciferase reporter plasmids containing different lengths of promoter fragments were constructed. Luciferase activities in breast cancer cells transiently transfected with these reporter plasmids were analyzed to examine the effects of PRRG4 overexpression on promoter activity. Transwell assays were performed to determine the effects of PRRG4-regulated pathway on migratory behaviors of breast cancer cells.

**Results:**

Analysis of the RNA-seq data revealed that PRRG4 knockdown decreased the transcript levels of all the mitochondrial protein-encoding genes. Subsequently, studies with PRRG4 knockdown and overexpression showed that PRRG4 expression increased mitochondrial DNA (mtDNA) content. Mechanistically, PRRG4 via Src activated STAT3 in breast cancer cells. Activated STAT3 in turn promoted the transcription of mtDNA polymerase *POLG* through a STAT3 DNA binding site present in the *POLG* promoter region, and increased mtDNA content as well as mitochondrial ATP production and oxygen consumption. In addition, PRRG4-mediated activation of STAT3 also enhanced filopodia formation, migration, and invasion of breast cancer cells. Moreover, PRRG4 elevated migratory behaviors and mitochondrial function of breast cancer cells through POLG.

**Conclusion:**

Our results indicate that PRRG4 via the Src-STAT3-POLG axis enhances mitochondrial function and promotes migratory behaviors of breast cancer cells.

**Supplementary Information:**

The online version contains supplementary material available at 10.1186/s12935-023-03178-0.

## Introduction

Breast cancer is the most common malignancy among women worldwide [1]. Despite great efforts to enhance breast cancer early detection and develop treatments for breast cancer, resistance to therapies and metastasis remain the major causes of mortality for breast cancer patients [2, 3]. Therefore, detailed investigation to understand the molecular mechanisms of metastasis should help uncover potential targets for the treatment of metastatic breast cancer.

PRRG4, a member of the proline rich γ-carboxyglutamine (PRRG) protein family, can interact via its intracellular PY motifs with WW domain-containing proteins that may mediate the biological function of PRRG4 [4–6]. PRRG4 has been implicated in the Wilms tumor, Aniridia, Genitourinary anomalies, and mental Retardation (WAGR) syndrome due to genetic linkage studies [7, 8]. Although the in vivo function of PRRG4 is still not understood, a study of the PRRG4 functional homolog Comm in Drosophila suggests that PRRG4 may play a role in guiding long range axon extension of specific neurons during embryonic development [9]. Recent studies have begun to reveal the important roles of PRRG4 in cancer progression [10, 11]. We have shown recently that PRRG4 promotes the migration, invasion, and metastasis of breast cancer cells by binding the E3 ubiquitin ligase NEDD4 via its PY motifs, degradating Robo1, and activating FAK and Src [10]. However, it is still less clear how activation of FAK-Src signaling by PRRG4 contributes to breast cancer metastasis.

The signal transducers and activators of transcription (STAT) factors can be activated by numerous signaling molecules beside JAKs [12, 13] and participate in a variety of biological functions including cell differentiation, proliferation, and survival [14, 15]. Among the STAT family members, STAT3 is a key molecule in development and carcinogenesis [16, 17]. STAT3 is constitutively activated during various stages of breast carcinogenesis including metastasis [18]. Previous studies showed that STAT3 is persistently activated by Src in cells stably transformed by the oncogene Src [19]. In addition, STAT3 is a required downstream effector of Src to induce podosome structures and related invasive phenotypes, suggesting that Src-STAT3 is one of the signaling pathways mediating cancer progression [20].

Mitochondria contain their own genome, mitochondrial DNA (mtDNA), which encodes 13 polypeptides of the oxidative phosphorylation (OXPHOS) system in the respiratory chain important for ATP production [21]. Accumulating evidence has indicated that mitochondria also play crucial roles in various aspects of metastasis including cancer cell motility, invasion, and colonization [22–24]. A published study has revealed that pseudopodia at the leading edges of migrating cells are the direct driving force for cancer cell migration, which requires an abundance of ATP [25]. In this study, through a transcriptome analysis of breast cancer cells with PRRG4 knockdown by PRRG4 shRNAs, we uncovered a link between PRRG4 and cell motility through mitochondria in breast cancer cells. PRRG4 regulated mtDNA content and mitochondrial function through activation of STAT3. Our results reveal that PRRG4 promotes migration and invasion of breast cancer cells by modulating mitochondrial function through the Src-STAT3-POLG axis.

## Materials and methods

### Cell culture

Breast cancer cell lines, SKBR3, MDA-MB-231 and HCC1954, were obtained from the American Tissue Culture Collection (ATCC). SUM149PT cell lines were purchased from Asterand Bioscience (Detroit, Michigan, USA). SKBR3 cells were cultured in DMEM medium (Gibco, California, USA) with 5% fetal bovine serum (FBS) (Lonsera, Australia) supplemented with 100 units/mL penicillin and 100 µg/mL streptomycin (Beyotime Biotechnology, Jiangsu, China). MDA-MB-231 cells were cultured in MEM medium (Gibco) containing 5% FBS, 1.8 µg/mL insulin (Solarbio, Beijing, China), 100 units/mL penicillin, and 100 µg/mL streptomycin. HCC1954 cells were cultured in RPMI 1640 medium (Gibco) containing 10% FBS, 100 units/mL penicillin, and 100 µg/mL streptomycin. SUM149PT cells were cultured in DMEM/F-12 with 5% FBS, 1 µg/mL hydrocortisone, 5 µg/mL insulin, 100 units/mL penicillin, and 100 µg/mL streptomycin. Cells were incubated in tissue culture incubators with an atmosphere of 5% CO2. Breast cancer cell lines used in this study were authenticated in 2020 using short tandem repeat analysis by Genetic Testing Biotechnology (Suzhou, China), and tested for mycoplasma using Myco-Lumi™ Luminescent Mycoplasma Detection kit (Beyotime Biotechnology) every six months.

### Plasmids

Retroviral pBabe-puro-PRRG4-WT and pBabe-puro-PRRG4-Y189A plasmids, and lentiviral PLKO1 plasmids containing two different PRRG4 shRNAs (#sh94 and #sh95) were generated as described previously [10]. The pGL3-basic luciferase reporter plasmid (pGL3-luc) was a kind gift from Dr. Haishan Huang (Wenzhou Medical University, Wenzhou, China). pGL3-luc plasmids containing different lengths of *POLG* promoter regions were generated in the following way. PCR fragments spanning − 1516 to + 155 bp and − 994 to + 155 bp of *POLG* promoter region were subcloned into the pGL3-luc, generating pGL3-(− 516/+ 155)-luc and pGL3-(− 994/+ 155)-luc. pGL3-luc plasmids containing the *POLG* promoter regions with the two predicted STAT3 binding site mutated, pGL3-(− 1516/+ 155)-Mut1 and pGL3-(− 1516/+ 155)-Mut2, were generated from pGL3-(− 1516/+ 155)-luc as a template using site-directed mutagenesis PCR. Primer sequences for subcloning and site mutagenesis were shown in Additional file 1. All the generated plasmids were verified by DNA sequencing (Tsingke, Beijing, China). Lentiviral plasmid PLEX expressing dominant negative (DN) STAT3 Y705F was a kind gift from Dr. Bolin Liu (Louisiana State University Health Sciences Center, USA).

### Antibodies and reagents

The anti-β-actin (cat#3700), anti-STAT3 (cat#9139), anti-phospho-STAT3 (Tyr705) (cat#9145), anti-Src (cat#2109), and anti-phospho-Src (Tyr416) (cat#2101) antibodies were from Cell Signaling Technology. Anti-PRRG4 (cat#HPA009040) antibodies were from Sigma. Anti-POLG (cat#ab128899) antibodies were from Abcam. STAT3 inhibitor Stattic and Src inhibitor Dasatinib were purchased from MedChemExpress. Actin-Tracker Green (cat#C1033) was from Beyotime Biotechnology.

### Retrovirus and lentivirus production, and viral Infection

The production of retroviruses and lentiviruses as well as viral infection of cells were performed essentially as described [26, 27].

### RNA-seq and data analysis

Total RNAs were isolated from SKBR3 cells expressing control-sh, PRRG4-sh94, and PRRG4-sh95, and subjected to RNA-seq analysis by Novogene company (Beijing, China). RNA integrity was evaluated using the RNA Nano 6000 Assay Kit of the Bioanalyzer 2100 system (Agilent Technologies, CA, USA). The DESeq2 R software was used to analyze the differential expression analysis (1.20.0). The resulting P-values were adjusted using the Benjamini and Hochberg’s method for controlling false discovery rate. Genes identified by DESeq2 with an adjusted p-value < 0.05 were assigned as differentially expressed.

### Quantitative PCR (q-PCR) analysis

Total RNAs were isolated from cells using the TRIZOL reagent (Invitrogen, USA) and reverse-transcribed into cDNA using HiScript II Q RT SuperMix (Vazyme, Jiangsu, China). q-PCR analysis was used to quantify the relative levels of mitochondrial DNA-encoded transcripts and mitochondrial DNA polymerase *POLG* using gene specific primers and ChamQ Universal SYBR Qpcr Master Mix (Vazyme). *β*-actin was used as an internal control. The primers sequences for q-PCR are shown in Additional file 1.

### Analysis of mitochondrial DNA (mtDNA) content

Genomic DNA was extracted from cells using a standard SDS lysis protocol as previously described [28]. Quantitative PCR was used to quantify the relative mtDNA content. The 16S ribosomal RNA gene in mtDNA was used to quantify the relative mtDNA content. The 18S rDNA in nuclear DNA was used as an internal control. The primer sequences are shown in Additional file 1.

### Analysis of cellular ATP contents

Cells (1 × 10^6^) were washed with cold phosphate-buffered saline (PBS) and lysed by boiling in buffer (100 mM Tris, 4 mM EDTA, pH 8.0) for 90 s. The supernatant was obtained by centrifugation at 10,000×*g* for 1 min, and subjected to ATP content analysis using the ATP measurement kit (ThermoFisher Scientific, USA) and Varioskan™ Flash Multimode Reader (Thermo Scientific). To measure mitochondrial ATP synthesis, the cells were pretreated with 1 µg/mL oligomycin for 1 h at 37 ℃ before lysis.

### Western blot

Whole-cell lysates were generated by lysing breast cancer cells in 1× SDS sample buffer (62.5 mM Tris-HCl pH 6.8, 2% SDS, 10% glycerol, 0.002% bromophenol blue, and 5% β-mercaptoethanol). Equal amounts of cell lysates were resolved on 10% SDS-PAGE and transferred to PVDF membranes (Millipore, USA). The membranes were blocked in 5% nonfat dry milk, incubated with the appropriate primary and horseradish peroxidase (HRP)-conjugated secondary antibodies, and developed using enhanced chemiluminescence with images captured by the ChemiDoc MP imaging system (Bio-rad, USA). Densitometry was used to quantify bands in western blots from three independent experiments, which were normalized to *β*-actin, and presented as fold changes relative to control that was set to1.

### Oxygen consumption measurement

Oxygen consumption rate (OCR) was measured with an oxygen electrode system Oxygraph-2k (Oroboros, Austria). Cells were washed with PBS and TDS (10 mM KCl, 0.137 M NaCl, 0.7 mM Na2HPO4, 10% calf serum, and 25 mM Tris-HCl pH 7.4–7.5), and then resuspended in TD (10 mM KCl, 0.137 M NaCl, 0.7 mM Na2HPO4, and 25 mM Tris-HCl, pH 7.4–7.5). To measure the basal respiratory oxygen consumption, cells (1 × 10^6^) were added to the test chamber. Addition of oligomycin (0.08 µg/mL, Sigma) was used to measure the level of oxygen consumption after inhibition of mitochondrial ATP synthase, and the subsequent addition of FCCP (0.08 µM, Sigma) was used to measure the maximum oxygen consumption.

### Luciferase assay

MDA-MB-231 cells with vector alone or PRRG4 overexpression, and SUM149PT cells with vector or STAT3-DN overexpression were seeded in 24-well plates for 24 h followed by being co-transfected with pGL3-*POLG* promoter-luciferase plasmids and pRL-TK-renilla luciferase plasmid (used as an internal control for transfection efficiency) (pGL3-basic plasmid: pRL-TK-renilla plasmid = 20:1). Forty-eight hours post-transfection, luciferase activity in the transfected cells was analyzed using the Dual-Luciferase Report Assay kit (Promega, USA) according to the manufacturer’s instructions.

### Analysis of filopodia

MDA-MB-231 and HCC1954 cells were serum-starved for 20 h, and stimulated with DMEM containing 10% FBS for 1 h. Cells were fixed with 3.7% formaldehyde, permeabilized with 0.1% Triton X-100, washed, and incubated with Actin-Tracker Green (Beyotime Biotechnology), which is phalloidin labeled with FITC. Images of stained cells were captured by a fluorescent confocal microscope (Nikon A1, Japan). Filopodia were identified as slender cytoplasmic protrusions supported by F-actin bundles present in the leading edge of migrating cells. Their lengths were determined with FiloQuant (http://imagej.net/FiloQuant).

### Cell migration and invasion assays

Transwell assays were used to assess the migration and invasion of breast cancer cells. Six hundred-microliter of DMEM containing 20% FBS was added into the lower compartment of the transwell, and cells (1.5 × 10^4^) resuspended in 200 µL serum‐free DMEM were seeded into the upper chamber. For invasion assay, transwell inserts were coated with 40 µL Matrigel (Corning, USA), and incubated overnight at 37 °C before cell seeding. After incubation for 20 h, cells in the lower compartment were fixed and stained with Hoechst dye (5 µg/mL) (Invitrogen), visualized under fluorescent microscope, and counted.

### Statistical analysis

Statistical analyses were performed by using GraphPad Prism 7. Student’s t-test and ANOVA were used to analyze the statistical significance of paired samples and multi-group samples, respectively. *p* < 0.05 was indicated statistically significant. All experiments were repeated at least three times with similar results.

## Results

### PRRG4 knockdown decreases the transcripts of mitochondrial DNA-encoded genes

**Fig. 1:**
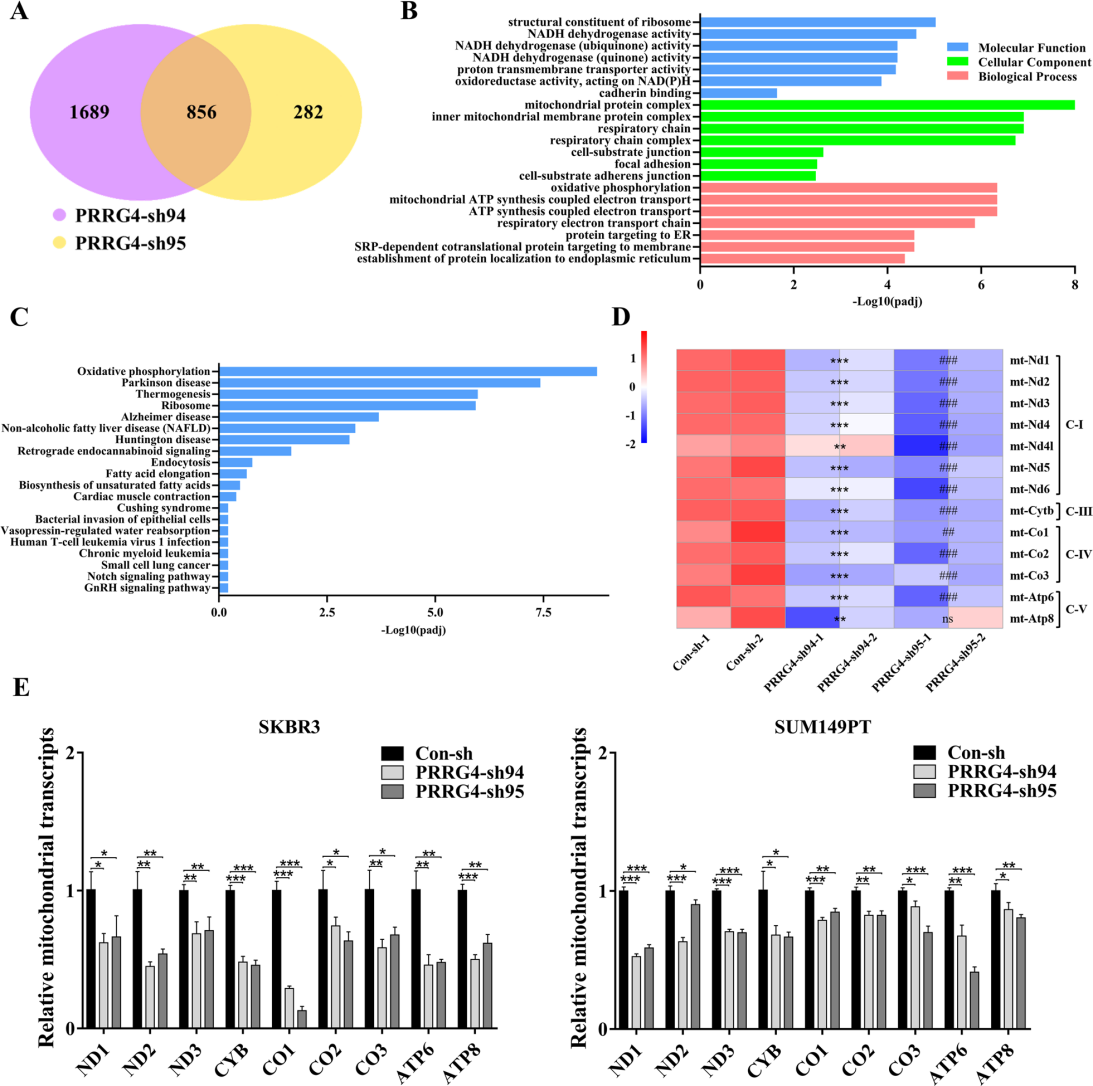
Knockdown of PRRG4 decreases the mRNA levels of mtDNA protein-encoding genes. **A** A Venn diagram showing the overlap of DEGs between SKBR3 cells expressing two different PRRG4-shRNAs (sh94 and sh95) compared with the control-shRNA. **B**, **C** GO and KEGG analyses demonstrated that DEGs were significantly enriched in electron transport chain and oxidative phosphorylation. **D** Heat map of RNA-seq data showing the mRNA levels of mitochondrial protein-encoding genes in SKBR3 cells expressing two different PRRG4-shRNAs (sh94 and sh95) and control-shRNA. PRRG4-sh94 group compared with control-shRNA group, **p < 0.01, ***p < 0.001; PRRG4-sh95 group compared with control-shRNA group, ^##^*p* < 0.01, ^###^*p* < 0.001, ns *p* > 0.05. **E** PRRG4 knockdown reduces the transcript levels of mitochondrial protein encoding genes. Total RNAs were isolated from SKBR3 and SUM149PT cells expressing control-shRNA and two different PRRG4-shRNAs (sh94 and sh95), reverse-transcribed, and subjected to q-PCR to quantitate the relative levels of transcripts for the indicated mtDNA-encoded genes. *p < 0.05, **p < 0.01, ***p < 0.001

To explore the molecular mechanism of PRRG4 action in breast cancer, we performed RNA-seq of SKBR3 cells expressing control-shRNA and two different PRRG4-shRNAs. Differentially expressed genes (DEGs) were analyzed in cells with PRRG4-shRNAs in comparison to control-shRNA with |logFC| > 0 and p ≤ 0.05. A total of 856 common DEGs were found between the two different PRRG4-shRNA groups (Fig. 1A).

To uncover the underlying interplay of these DEGs, GO and KEGG pathway enrichment analyses were performed. GO analysis revealed that the top DEGs were mainly enriched in mitochondrial structure and function, such as electron transport chain and oxidative phosphorylation (Fig. 1B). Similarly, KEGG pathway analysis found that the top DEGs were significantly enriched in oxidative phosphorylation (Fig. 1C). Further analysis revealed that the DEGs contained the 13 mitochondrial DNA (mtDNA) protein-encoding genes (Fig. 1D). Quantitative-PCR (qPCR) was used to verify this observation in two different breast cancer cell lines SKBR3 and SUM149PT with PRRG4 knockdown. In our previous published work, these two cell lines expressed higher level of endogenous PRRG4 protein [10]. The analysis showed that the RNA contents of the majority of mtDNA protein-encoding genes were significantly lower in cells expressing two different PRRG4-shRNAs compared with cells expressing control-shRNA (Fig. 1E).

### PRRG4 knockdown decreases mtDNA content and impairs mitochondrial function

**Fig. 2:**
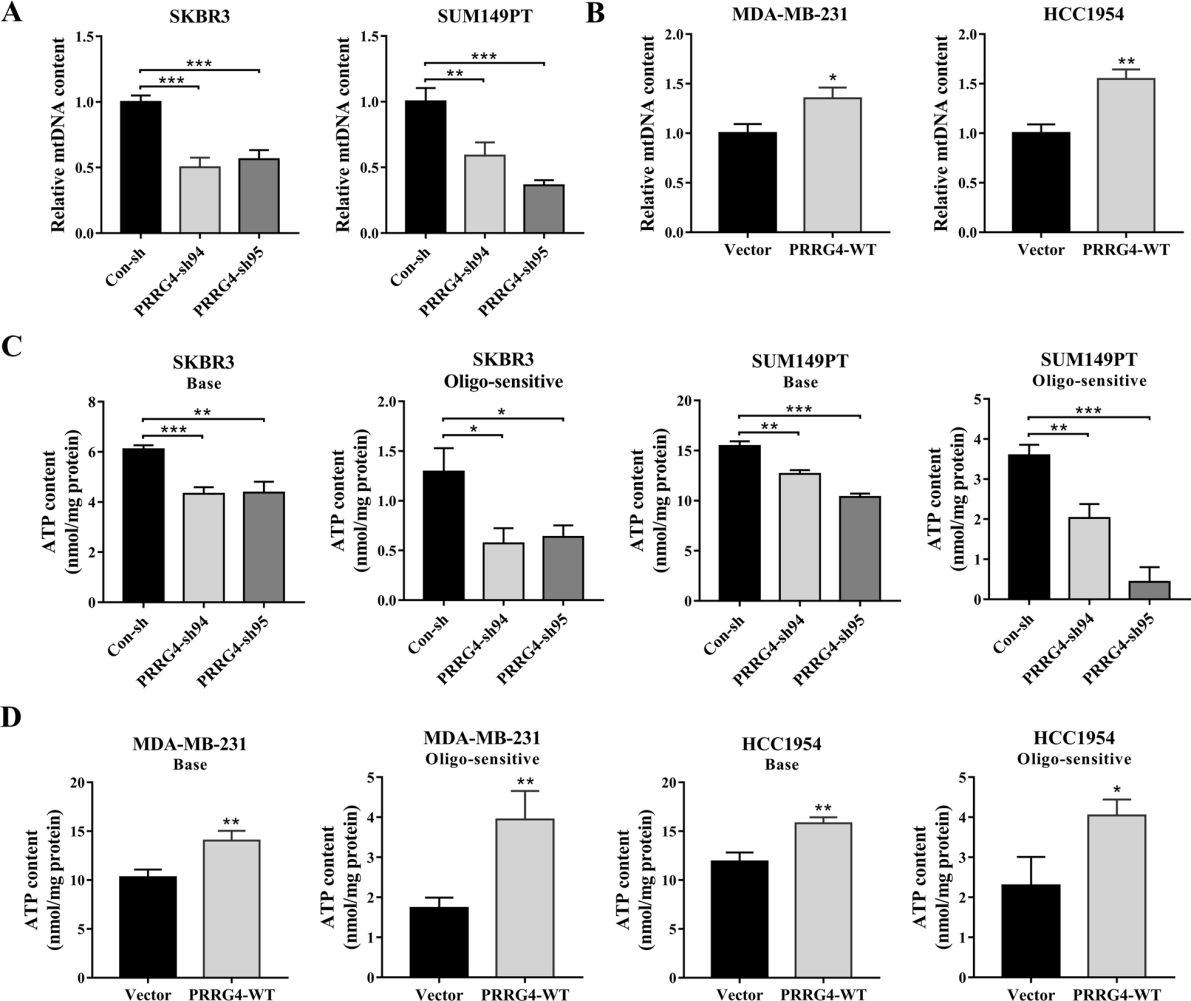
PRRG4 regulates mtDNA content and mitochondrial function. **A** Knockdown of PRRG4 with shRNAs decreases mtDNA content in SKBR3 and SUM149PT cells. **B** Overexpression of PRRG4 increases mtDNA content in MDA-MB-231 and HCC1954 cells. **C** and **D** The basal ATP content (Base) and oligomycin-sensitive ATP content (Oligo-sensitive) in SKBR3 and SUM149PT with PRRG4 knockdown, and MDA-MB-231 and HCC1954 with PRRG4 overexpression were measured using a luciferin/luciferase chemiluminescence ATP assay kit. Cells were treated with 1 µg/mL oligomycin for 1 h. *p < 0.05, **p < 0.01, ***p < 0.001

Since the transcripts of most of mtDNA-encoded genes were reduced by PRRG4 knockdown, we suspected that PRRG4 may regulate mtDNA content in breast cancer cells. We assessed the relative mtDNA content in breast cancer cells using qPCR analysis. The result showed that mtDNA content was significantly reduced in SKBR3 and SUM149PT cells expressing PRRG4-shRNAs in comparison to cells expressing control-shRNA(Fig. 2A). In contrast, in MDA-MB-231 and HCC1954 cells, two cell lines with low level of endogenous PRRG4 [10], mtDNA content was notably increased in cells overexpressing PRRG4 compared with the respective vector control cells (Fig. 2B). These data suggest that PRRG4 mainly controls mtDNA-encoded gene expression mainly through regulating mtDNA content.

Since mtDNA-encoding proteins are key components of the mitochondrial respiratory chain complexes that are involved in oxidative phosphorylation and cellular energy production, their decreased expression should affect mitochondrial function including ATP production. We examined ATP content using a luciferin/luciferase chemiluminescence ATP assay kit. Knockdown of PRRG4 decreased the basal ATP content in SKBR3 and SUM149PT cells (Fig. 2C, base), whereas overexpression of PRRG4 increased the ATP content in MDA-MB-231 and HCC1954 cells (Fig. 2D, base). Importantly, ATP production from mitochondria, which was reflected as the reduced ATP content in response to oligomycin (ATP synthase inhibitor) treatment (oligomycin-sensitive), was significantly decreased in cells with PRRG4 knockdown (Fig. 2C, oligo-sensitive) but increased in cells with PRRG4 overexpression (Fig. 2D, oligo-sensitive). These results show that PRRG4 expression regulates mtDNA content and mitochondrial function in breast cancer cells.

### PRRG4 promotes STAT3 activation via src

In our published study, we showed that PRRG4 can interact with and activate protein tyrosine kinase Src [10]. To understand how PRRG4 regulates mtDNA content, we asked whether Src may involve in this process. Considering the reported roles of STAT3 in mitochondrial function [29] and oxidative phosphorylation [30], we wanted to test whether PRRG4 can activate STAT3 via Src especially considering that Src and STAT3 are commonly involved in breast cancer [31, 32].

**Fig. 3:**
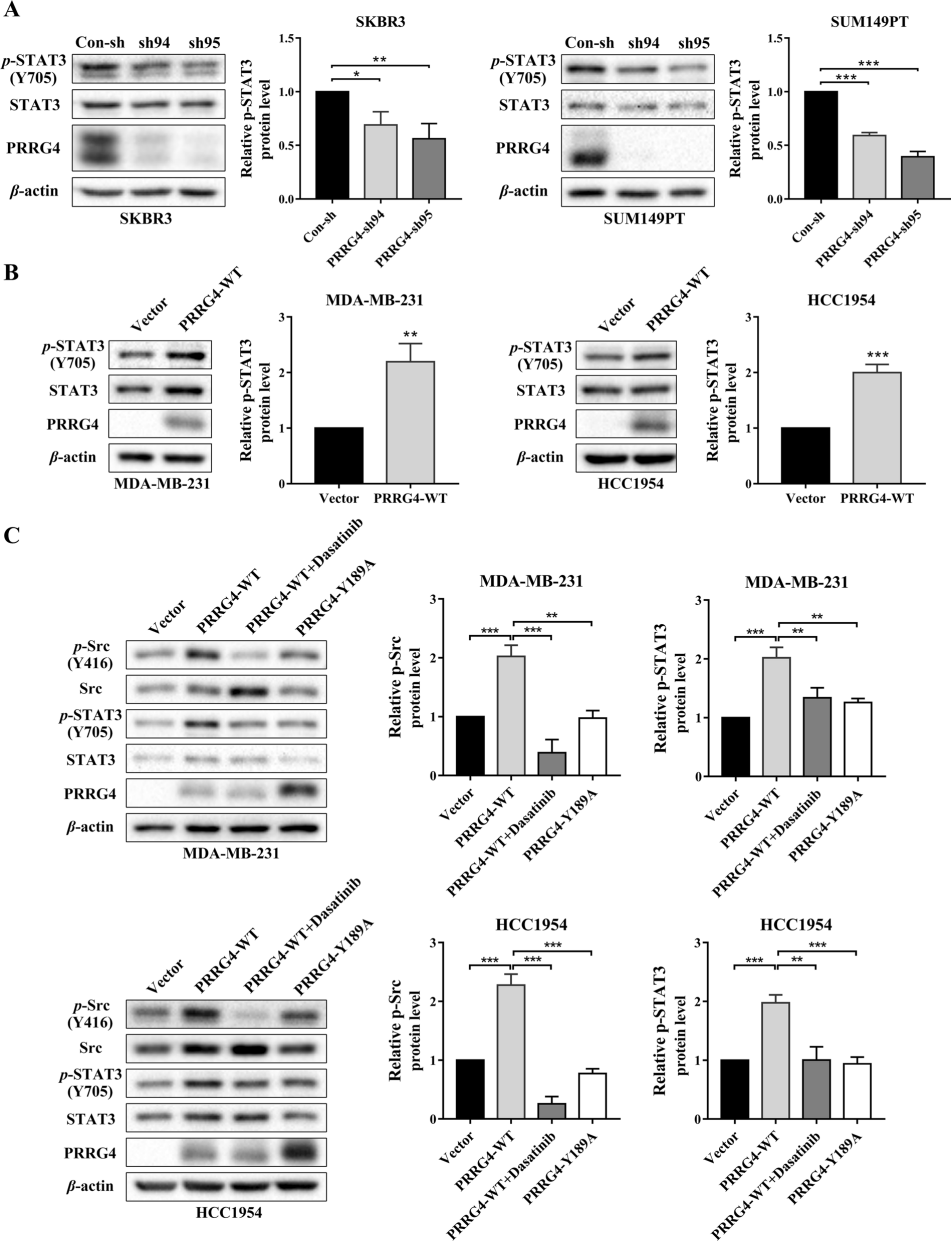
PRRG4 enhances STAT3 activation via Src. **A** Knockdown of PRRG4 decreases STAT3 activation in breast cancer cells. **B** PRRG4 overexpression enhances STAT3 activation in breast cancer cells. **C** PRRG4 via its WW domain binding motif LPSY189 promotes STAT3 activation through Src activation in breast cancer cells. MDA-MB-231 cells with vector, PRRG4-WT, and PRRG4-Y189A mutant were treated with vehicle or Src inhibitor Dasatinib (40 nM for MDA-MB-231, and 80 nM for HCC1954) for 2 h before being subjected to immunoblotting. p-Stat3 and p-Src levels were quantified and normalized to corresponding β-actin levels, which were presented as fold changes relative to controls that were set to 1. *p < 0.05, **p < 0.01, ***p < 0.001

To investigate this possibility, we examined the effect of PRRG4 knockdown on STAT3 activation in breast cancer cells by immunoblotting with phospho-STAT3 (Tyr705) antibody (p-STAT3). Knockdown of PRRG4 significantly decreased p-STAT3 in both SKBR3 and SUM149PT cells compared with knockdown control (Fig. 3A). Conversely, p-STAT3 levels were also examined in MDA-MB-231 and HCC1954 cells with retroviruses expressing vector alone and PRRG4-wild type (WT). Compared with vector only, PRRG4-WT enhanced p-STAT3 levels in breast cancer cells (Fig. 3B). To test whether PRRG4 activation of STAT3 is mediated by Src, p-STAT3 levels were examined in PRRG4-WT cells treated with Dasatinib, a Src inhibitor, and in cells expressing PRRG4 point mutant Y189A. Our published data showed that PRRG4-Y189 is important for activation of Src [10]. In both cell lines, Dasatinib treatment prevented the increase in p-STAT3 level induced by PRRG4-WT (Fig. 3C, lane 3). Likewise, PRRG4-Y189A failed to enhance pSTAT3 level and Src phosphorylation compared with PRRG4-WT (Fig. 3C, lane 4). These results indicate that PRRG4 enhances STAT3 activation via Src.

### PRRG4 regulates mtDNA content and mitochondrial function through activation of STAT3

**Fig. 4:**
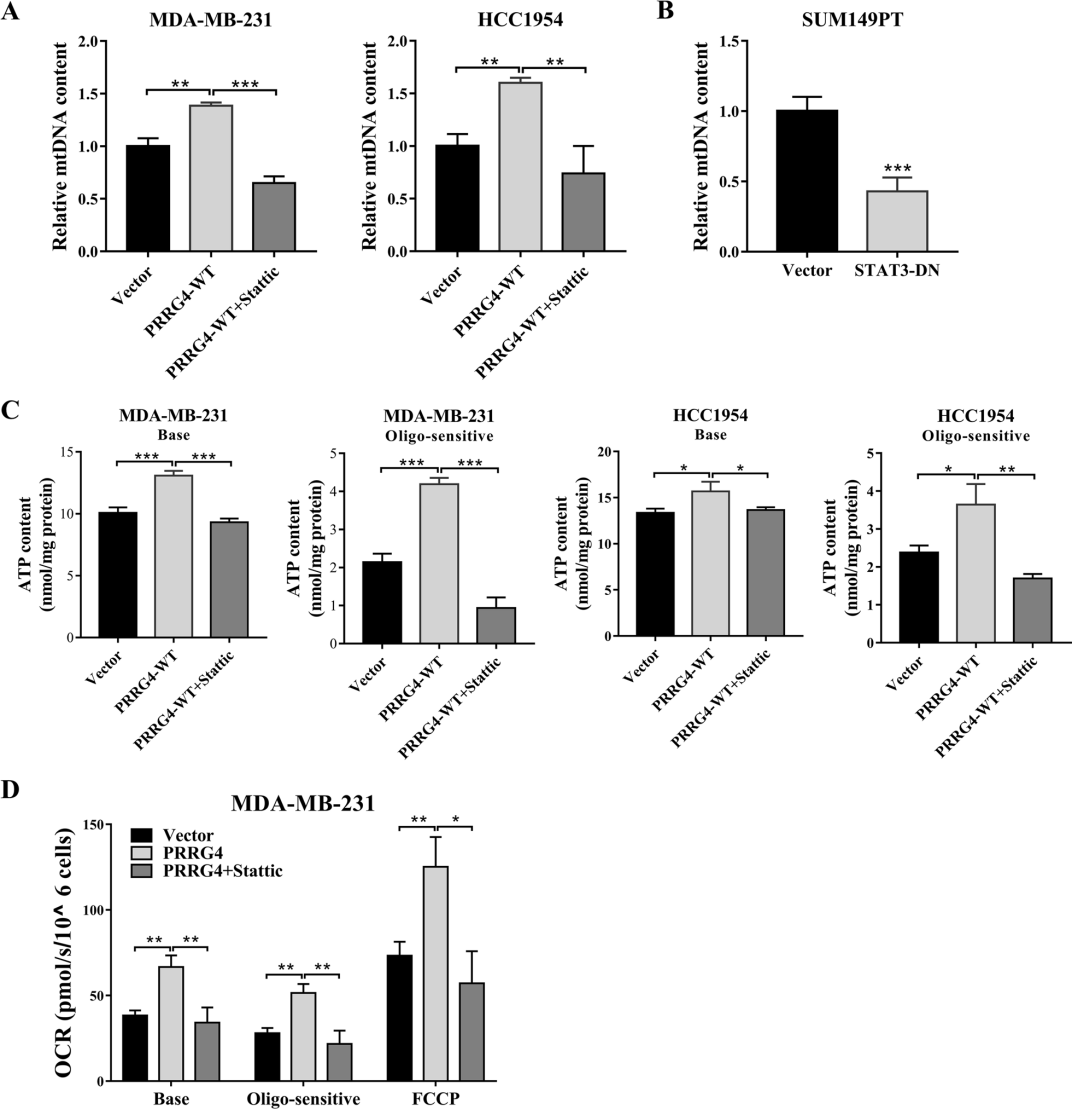
PRRG4 regulates mtDNA content and mitochondrial function through STAT3. **A** Stattic decreases mtDNA content enhanced by PRRG4 overexpression in breast cancer cells. Cells with PRRG4 overexpression were treated with vehicle or Stattic (3 µM for MDA-MB-231, and 2 µM for HCC1954 cells) for 20 h before being subjected to qPCR analysis for mtDNA content. **B** Overexpression of a dominant negative STAT3 mutant (STAT3-DN) decreases mtDNA content in SUM149PT cells. **C** Overexpression of PRRG4 increases the basal ATP (Base) and oligomycin-sensitive ATP (Oligo-sensitive) contents through STAT3. Cells with PRRG4-WT were treated with vehicle or Stattic (3 µM for MDA-MB-231, and 2 µM for HCC1954 cells) for 20 h. Cells were treated with 1 µg/mL oligomycin for 1 h. **D** Overexpression of PRRG4 increases the basal, ATP-linked respiration (oligo-sensitive), and maximum respiratory capacity (FCCP) through STAT3. MDA-MB-231 cells were treated with vehicle or Stattic (3 µM) for 20 h. The basal, ATP-linked respiration, and maximum respiratory capacity were calculated in response to treatment with oligomycin (0.08 µg/mL) and FCCP (0.08 µM), respectively. *p < 0.05, **p < 0.01, ***p < 0.001

To detect whether STAT3 mediates PRRG4-enhanced mtDNA and ATP contents in breast cancer cells, we examined the effects of Stattic (a STAT3 inhibitor) treatment on MDA-MB-231 or HCC1954 cells overexpressing PRRG4-WT. Compared with vector control, PRRG4-WT expression enhanced mtDNA contents, but failed to increase mtDNA levels in the presence of Stattic in both cell lines (Fig. 4A). To further collaborate the involvement of STAT3, we also examined the effects of expressing a dominant negative form of STAT3 (STAT3-DN) in SUM149PT cells that express higher level of endogenous PRRG4. Compared with vector control, mtDNA content was significantly decreased in cells with STAT3-DN (Fig. 4B). In addition, while PRRG4-WT expression increased the basal and oligomycin-sensitive ATP contents compared with vector control, addition of Stattic blocked PRRG4-WT-enhanced ATP levels in both breast cancer cell lines (Fig. 4C). Furthermore, we also examined the effect of PRRG4 on oxygen consumption in the absence or presence of oligomycin and FCCP (mitochondrial oxidative phosphorylation uncoupler). Our results showed that the basal, ATP-linked respiration (oligo-sensitive), and maximum respiratory capacity (FCCP) were significantly increased in cells with PRRG4 overexpression compared with the vector control. The addition of Stattic blocked the increases in three types of oxygen consumption induced by PRRG4 overexpression (Fig. 4D). Together, these findings demonstrate that PRRG4 increases mtDNA content and promotes mitochondrial function through activation of STAT3 in breast cancer cells.

### PRRG4 promotes the expression of mitochondrial DNA polymerase *POLG *through STAT3

**Fig. 5:**
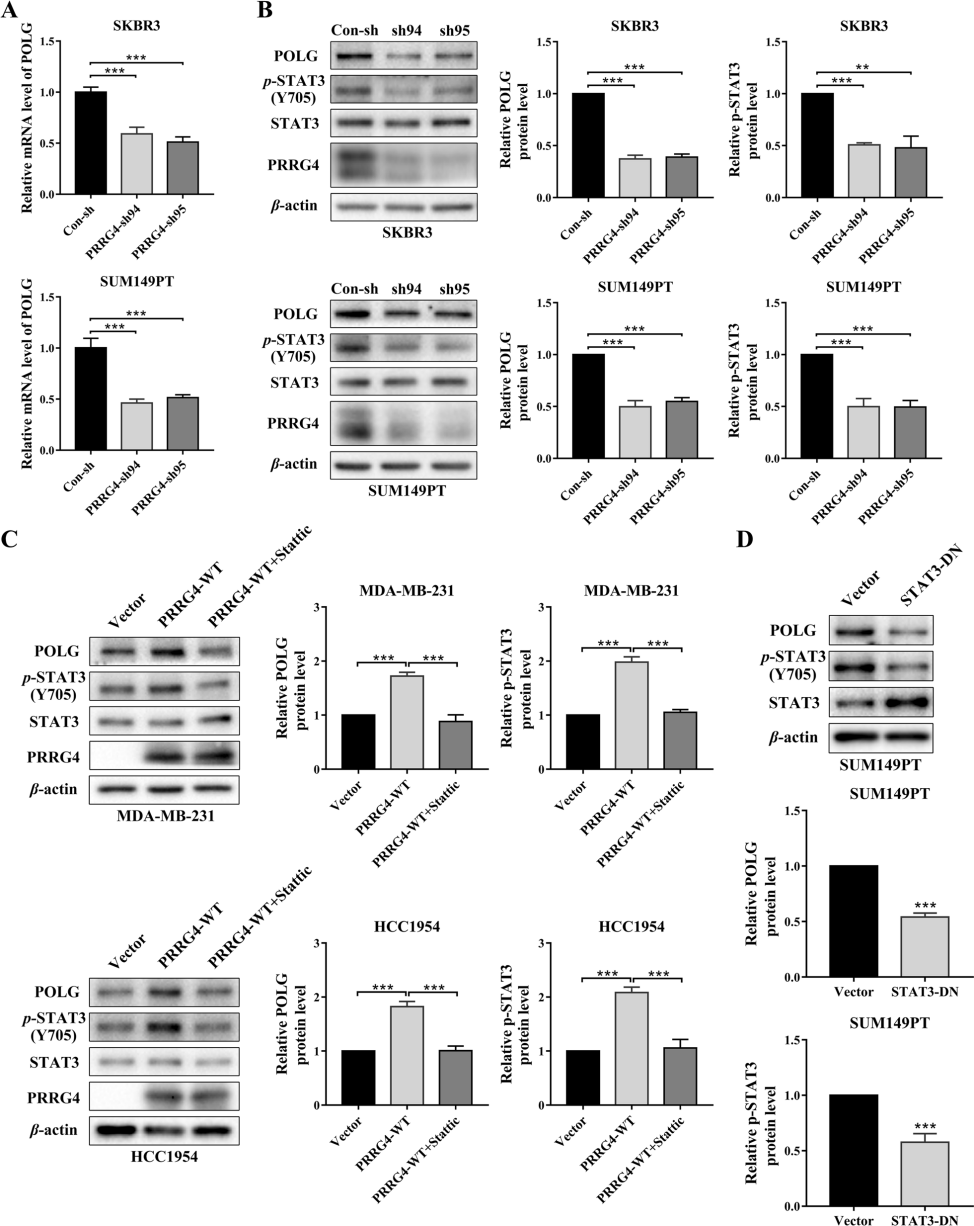
PRRG4 regulates the expression of mitochondrial DNA polymerase POLG through STAT3. **A** Knockdown of PRRG4 reduces the mRNA level of *POLG* in SKBR3 and SUM149PT cells. Relative mRNA levels of *POLG* were analyzed by qRT-PCR. **B** Knockdown of PRRG4 inhibits POLG protein levels in SKBR3 and SUM149PT cells. **C** PRRG4 overexpression enhances POLG protein levels through STAT3 activation in MDA-MB-231 and HCC1954 cells. Cells with PRRG4-WT were treated with vehicle or Stattic (3 µM for MDA-MB-231, and 2 µM for HCC1954 cells) for 20 h. **D** Overexpression of a dominant negative STAT3 mutant (STAT3-DN) reduces POLG protein levels in SUM149PT cells. POLG and p-Stat3 levels were quantified and normalized to corresponding β-actin levels, which were presented as fold changes relative to controls that were set to 1. **p < 0.01, ***p < 0.001

As mitochondrial DNA polymerase gamma (POLG) is essential for mtDNA replication [33], we hypothesized that PRRG4 may regulate mtDNA content by controlling POLG expression through STAT3 in breast cancer cells. qPCR analysis demonstrated that both PRRG4-shRNAs strongly reduced *POLG* mRNA levels compared with control-shRNA in SKBR3 and SUM149PT cells (Fig. 5A). Furthermore, POLG protein was also notably reduced in SKBR3 and SUM149PT cells expressing PRRG4-shRNAs in comparison to cells expressing control-shRNA (Fig. 5B). Conversely, POLG protein level was significantly increased in MDA-MB-231 and HCC1954 cells overexpressing PRRG4 compared with vector control cells (Fig. 5C, lane 2). Furthermore, treatment with the STAT3 inhibitor Stattic prevented the increase in POLG protein level induced by PRRG4 (Fig. 5C, lane 3). In addition, interfering STAT3 activation by expressing the dominant negative STAT3 also reduced POLG protein levels in SUM149PT cells (Fig. 5D). Taken together, our results indicate that STAT3 mediates PRRG4-induced POLG expression in breast cancer cells.

### PRRG4 enhances *POLG *transcription via STAT3

**Fig. 6:**
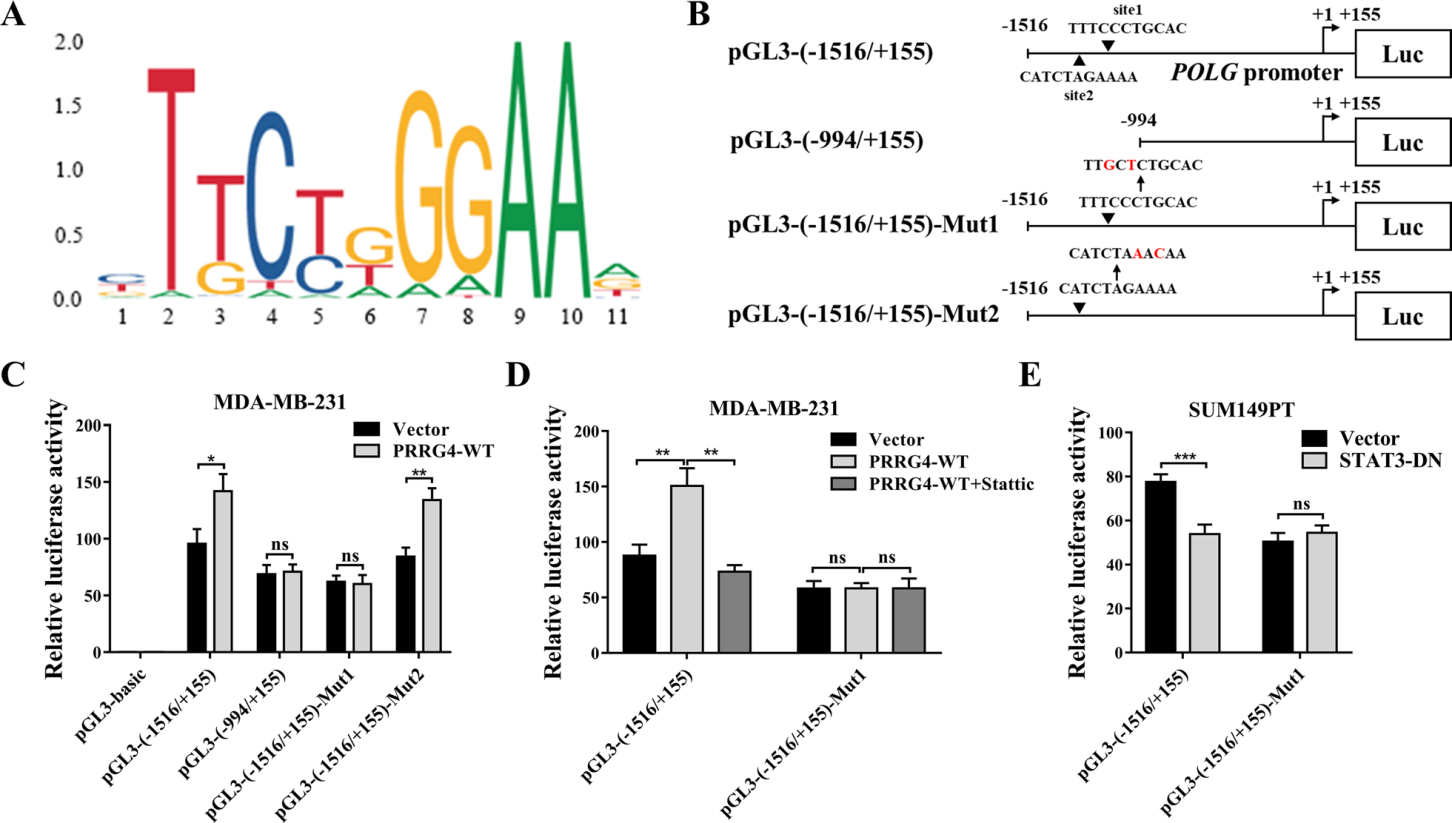
PRRG4 enhances *POLG* transcription via STAT3. **A** The consensus STAT3 DNA binding motif was downloaded from JASPAR. **B** Different pGL3-*POLG* promoter (up to − 1516 bp)-luciferase reporter (luc) constructs. Two predicted STAT3 binding sites, site1 and site2 in *POLG* promoter are indicated by arrowhead. Mut1 and Mut2 are corresponding site1 and site2 that were mutated respectively in positions indicated by red letters. **C** Luciferase reporter assay shows that predicted STAT3 binding site1 mediates PRRG4 enhancement of *POLG* promoter activity. The indicated luciferase reporter plasmids together with pRL-TK normalizing plasmid were transiently transfected into MDA-MB-231 cells with vector and PRRG4-WT. Forty-eight hours later, cells were subjected to dual-luciferase activity assay. **D** PRRG4 enhances *POLG* promoter activity depending on STAT3. The indicated luciferase reporter plasmids together with pRL-TK normalizing plasmid were transiently cotransfected into MDA-MB-231 cells with vector alone or PRRG4-WT. Twenty-eight hours later, MDA-MB-231 cells were treated with vehicle or Stattic (3 µM) for 20 h. **E** Expression of STAT3-DN decreases *POLG* promoter activity in SUM149PT cells. SUM149PT cells with vector alone and STAT3-DN were transiently cotransfected with the indicated luciferase reporter plasmids together with pRL-TK normalizing plasmid. *p < 0.05, **p < 0.01, ***p < 0.001, ns: no significance

Since STAT3 is a transcription factor, we hypothesized that STAT3 promotes *POLG* transcription in breast cancer cells. Analysis of DNA sequence in the 1.5 kb *POLG* promoter region using the online website JASPAR (http://jaspar.genereg.net/) revealed two potential STAT3 DNA binding sites, similar to the STAT3 binding motif (5′-TTCTGGGAA-3′) (Fig. 6A), are located between − 1516 to − 994 bp upstream of the *POLG* transcription start site (Fig. 6B). To test whether these two sites mediate PRRG4 enhancement of *POLG* expression, DNA fragments between − 1516 to + 155 bp and − 994 to + 155 bp relative to *POLG* transcription start site were inserted in front of the *luciferase* in the pGL3-Basic plasmid respectively (Fig. 6B). These *POLG* promoter luciferase reporter constructs were cotransfected with vector or PRRG4 overexpression plasmid into MDA-MB-231 cells. Analysis of luciferase activities showed that both − 1516 to + 155 and − 994 to + 155 DNA fragments can drive *luciferase* expression. However, PRRG4 overexpression only significantly further potentiated luciferase activity driven by the − 1516 to + 155 fragment, suggesting that the region between − 1516 and − 994 bp mediates the PRRG4 action (Fig. 6C). Since the two predicted STAT3 binding sites are located between − 1516 and − 994 bp (Fig. 6B, site1 and site2), we mutated each of the two sites in the luciferase plasmid pGL3-(− 1516/+ 155), generating pGL3-(− 1516/+ 155)-Mut1 and pGL3-(− 1516/+ 155)-Mut2 reporter plasmids. Mutation of site1 prevented the elevation of luciferase activity induced by PRRG4 overexpression. In contrast, mutation of site2 did not affect increased luciferase activity induced by PRRG4 (Fig. 6C). This result indicated that the predicted STAT3 binding site at site1 mediates PRRG4 enhancement of *POLG* promoter activity.

To further support that STAT3 mediates PRRG4 enhancement of *POLG* promoter activity, we examined the effect of Stattic treatment on pGL3-(− 1516/+ 155) or pGL3-(− 1516/+ 155)-Mut1 cotransfected with PRRG4-WT. We found that Stattic blocked increased luciferase activity induced by PRRG4-WT in the pGL3-(− 1516/+ 155) transfected cells but did not have any effect on luciferase activity in the pGL3-(− 1516/+ 155)-Mut1 and PRRG4-WT cotransfected cells (Fig. 6D). In addition, we examined the effect of STAT3-DN on *POLG* promoter activity by transfecting pGL3-(− 1516/+ 155) or pGL3-(− 1516/+ 155)-Mut1 into SUM149PT cells expressing vector alone or STAT3-DN, respectively (Fig. 6E). The result showed that STAT3-DN blocked luciferase activity in pGL3-(− 1516/+ 155) transfected cells, but no effect on luciferase activity in pGL3-(− 1516/+ 155)-Mut1 transfected cells. These results supported the notion that STAT3 mediates PRRG4 enhancement of *POLG* transcription by binding to the predicted STAT3 binding site1 in *POLG* promoter region in breast cancer cells.

### PRRG4 promotes migration and invasion of Breast cancer cells through the STAT3-POLG axis

Our published result showed that PRRG4 promotes the migration and metastasis of breast cancer cells [10]. Mitochondria play key roles in cell migration by providing ATP for actin cytoskeleton remodeling [34]. Therefore, we hypothesized that PRRG4 via the STAT3-POLG pathway enhances cell migration by modulating mitochondrial function.

**Fig. 7:**
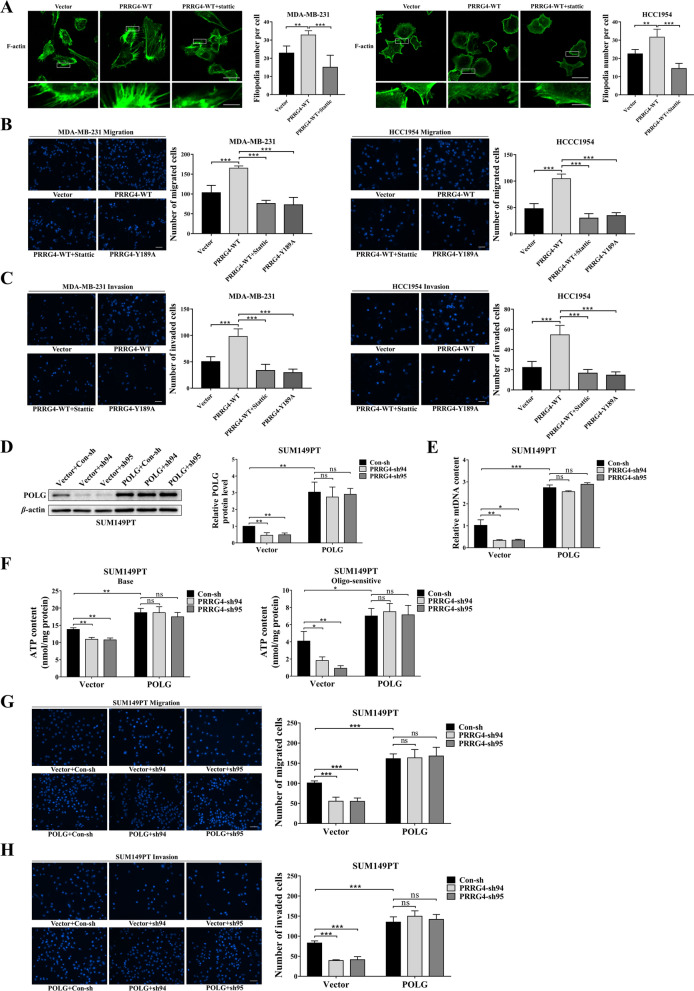
PRRG4 promotes migration and invasion of breast cancer cells through the STAT3-POLG axis. **A** STAT3 inhibitor decreased the filopodia formation enhanced by PRRG4 overexpression. Serum-starved cells seeded on cover slips were incubated with vehicle or 4 µM Stattic for 11 h, and stimulated with 10% FBS for 1 h in the presence of vehicle or 4 µM Stattic. Cells (MDA-MB-231 cells, left image panel; HCC1954 cells, right image panel) were fixed and stained with Actin-Tracker Green. Five micrographs/cell line were used to quantitate filopodia numbers. Scale bars, 25 μm (upper image panels), and 5 μm (lower image panels). **B** and **C** PRRG4 overexpression promotes migration and invasion of breast cancer cells through STAT3 and LPSY189 motif. Indicated cells were treated with vehicle or Stattic (3 µM for MDA-MB-231 and 2 µM for HCC1954) for 20 h. **D** Generation of cells with both POLG overexpression and PRRG4 knockdown. SUM149PT cells were infected with pBabe-Hygro vector alone and pBabe-Hygro POLG retroviruses, and selected with hygromycin before being infected with PLKO.1 lentiviruses expressing control-shRNA and two different PRRG4-shRNAs respectively. Cells were immunoblotted with the indicated antibodies. POLG levels were quantified and normalized to corresponding β-actin levels, which were presented as fold changes relative to control that was set to 1. **E** Overexpression of POLG increases mtDNA content and prevents the decrease in mtDNA content induced by PRRG4 knockdown. **F** Overexpression of POLG increases the basal (Base) and oligomycin-sensitive (Oligo-sensitive) ATP contents and prevents the decrease in the basal and oligomycin-sensitive ATP contents induced by PRRG4 knockdown. **G** and **H** POLG overexpression enhances cell migration and invasion, and prevents the decrease in cell migration and invasion induced by PRRG4 knockdown in SUM149PT cells. Scale bars, 50 μm *p < 0.05, **p < 0.01, ***p < 0.001

To begin to test this possibility, we investigated whether PRRG4 regulates the formation of filopodia, which plays a key role in driving cell migration in breast cancer cells [35, 36]. Compared with vector control, the number of filopodia per cell was dramatically increased in cells with PRRG4 overexpression. However, addition of STAT3 inhibitor Stattic blocked PRRG4-induced increase in filopodia (Fig. 7A). Next, we investigated whether STAT3 mediates PRRG4-enhanced migratory behaviors of breast cancer cells by performing transwell migration and invasion assays. Compared to vector control, PRRG4-WT significantly increased migration (Fig. 7B) and invasion (Fig. 7C) of MDA-MB-231 and HCC1954 cells. In contrast, treatment with Stattic prevented PRRG4-WT-induced increase in cell migration and invasion. Likewise, PRRG4-Y189A, which cannot enhance STAT3 phosphorylation (Fig. 3C), failed to enhance cell migration and invasion in comparison with vector control (Fig. 7B and C).

Subsequently, we wanted to examine whether POLG is involved in PRRG4’s action in enhancing breast cancer cell migration and invasion. To address this question, SUM149PT cells expressing control-shRNA and two different PRRG4-shRNAs were infected with retroviral vector alone or vector expressing POLG (Fig. 7D). qPCR was used to quantify mitochondrial DNA copy number in these cell lines (Fig. 7E). Similar to what we found earlier (Fig. 2A), knockdown of PRRG4 decreased mtDNA content. In contrast, overexpression of POLG not only increased mtDNA content but also prevented the decrease in mtDNA content induced by PRRG4 knockdown (Fig. 7E). Similarly, overexpression of POLG not only increased the basal and oligomycin-sensitive ATP contents but also prevented the decrease in the basal and oligomycin-sensitive ATP contents induced by PRRG4 knockdown (Fig. 7F). Furthermore, results from transwell assay also revealed that POLG overexpression enhanced cell migration and invasion (Fig. 7G and H), and rescued the reduced cell migration (Fig. 7G) and invasion (Fig. 7H) induced by PRRG4 knockdown in breast cancer cells. All these data indicate that PRRG4 via the STAT3-POLG pathway enhances the migration, invasion, and mitochondrial function of breast cancer cells. Our result is consistent with the idea that STAT3 regulates mitochondrial function through transcription regulation [37].

## Discussion

Metastasis is the leading cause of cancer deaths. Detailed understanding the mechanism governing the migratory behaviors of cancer cells should help develop new therapeutics to combat metastatic cancer. In this study, we uncover that PRRG4 via the Src-STAT3-POLG axis enhances mitochondrial DNA copy number and ATP production, and promotes the migration and invasion of breast cancer cells.

Our results reveal that PRRG4 plays an important role in regulating mtDNA content by increasing the expression of mitochondrial DNA polymerase POLG. PRRG4 knockdown leads to a prominent decrease in the mtDNA content in breast cancer cells, which likely contributes to the decrease in mRNA levels of the 13 mtDNA protein-encoding genes (Fig. 1D and E) and impaired mitochondrial functions (Figs. 2C and 4D) in PRRG4 knockdown cells. Numerous studies have suggested that alterations in mtDNA content affect tumorigenesis in a variety of human malignancies [38–40], although the upstream regulators of mtDNA content are still mostly unknown. For example, the invasive ability of mtDNA-depleted cells was notably increased through the mitochondrial stress-induced overexpression of cathepsin L and TGFβ1 [38]. In addition, loss of single strand DNA binding protein 1 (SSBP1) reduced mtDNA content, which enhanced calcineurin-mediated mitochondrial retrograde signaling that elevated TGFβ promoter activity and induced TGFβ-driven epithelial-to-mesenchymal transition of breast cancer cells [39]. Moreover, the increased mtDNA content in peripheral blood cells has been linked to the risk of developing a variety of tumors. Higher mtDNA content was linked to a higher risk of breast cancer, according to analysis of a single blood sample from patients obtained about 6 years before their cancer diagnosis [40].

Our study uncovers that PRRG4 via Src-STAT3 regulates POLG expression by increasing the transcription of *POLG* in breast cancer cells. Our previous study has shown that PRRG4 interacts with endogenous Src and enhances Src activation, which likely mediates PRRG4-induced migration and invasion of breast cancer cells [10]. Here, we found that PRRG4-WT, not PRRG4-Y189A (a mutant of PRRG4 unable to bind Src), promoted STAT3 activation (Fig. 3C). These data indicate that PRRG4 via its PY motifs stimulates Src that activates STAT3. Our data is consistent with previous finding that Src can promote STAT3 activation, which likely contributed to the tumorigenesis induced by Src [19]. Furthermore, our data showed that PRRG4 activated transcription factor STAT3, which enhanced *POLG* transcription (Fig. 6). Human mitochondrial DNA is replicated exclusively by the POLG, which is a nuclear-encoded, mitochondrial DNA replication enzyme that is crucial for the existence of eukaryotic life [41–44]. *POLG* homozygous knockout mice die in embryo due to a substantial mtDNA depletion and an early developmental abnormality [43]. Maintaining mtDNA content is crucial for mitochondrial energy generation because mtDNA encodes 13 proteins that, together with more than 85 nuclear-encoded proteins, are assembled to form the oxidative phosphorylation system [21]. It is reported that POLG mRNA was overexpressed in tumors from pancreatic ductal adenocarcinoma patients compared to normal controls [45]. In addition, shRNA-mediated knockdown of POLG decreased mtDNA content and increased the resistance to oxidative stress in tumoroids [46]. Moreover, POLG knockdown reduced OXPHOS activity and cellular glycolytic rate in gastric cancer cells [47]. These findings imply that POLG plays a significant role in tumorigenesis process. Our data showed that POLG overexpression rescued the decrease in cell motility, mtDNA content and ATP production (Fig. 7E–H) induced by PRRG4 knockdown. These results identify POLG as an important link between PRRG4 and breast cancer progression.

New therapeutic strategies for cancer are beginning to target the altered function of mitochondria. Recent studies have demonstrated the significance of mitochondrial-related metabolism mediated by fatty acids [48, 49], glutamine [50, 51] and pyruvate [52] in the course of metastatic disease. A published study demonstrated that suppression of PGC-1α, a critical regulator of mitochondrial biogenesis, reduces the invasion and metastasis of cancer cells. Circulating cancer cells use PGC-1α to enhance oxidative phosphorylation and oxygen consumption rate [53]. Pharmacological suppression of oxidative phosphorylation dramatically reduced metastatic seeding in the lungs, which demonstrated the functional significance of oxidative phosphorylation in metastasis [54]. Altering mitochondrial function may also affect other essential cellular functions through retrograde mitochondria-nucleus signaling and cause EMT-like reprogramming to a fibroblastic morphology and the acquisition of migratory and invasive phenotypes [55]. Our results demonstrate that STAT3 inhibition significantly reduced PRRG4-induced increase in oxygen consumption (Fig. 4D) and migratory behaviors of breast cancer cells (Fig. 7A–C). Therefore, PRRG4 via STAT3 likely promotes mitochondrial biogenesis and oxidative phosphorylation to enhance the migration of breast cancer cells. Further research is certainly required to specifically link PRRG4-induced oxidative phosphorylation and migratory behaviors of breast cancer cells. Our findings support the notion that migratory and invasive characteristics of cancer cells are dependent on mitochondrial function. In addition, the result of our study also suggests that *PRRG4*, a potential candidate gene for WAGR syndrome [7, 8], may regulate long range axon extension by modulating mitochondrial function during embryonic development.

## Conclusion

In summary, our study uncovers that PRRG4 promotes the invasion and metastasis of cancer cells by modulating mitochondrial function through the Src-STAT3-POLG axis. PRRG4 and PRRG4-regulated pathways should be potential treatment targets for metastatic breast cancer.

### Supplementary Information

**Additional file 1: Table S1.** Primer sequences for subcloning and point mutagenesis. **Table S2.** The sequences of the primers for q-PCR.

## Data Availability

The original data and materials used to support the findings of this study are available from the corresponding author upon reasonable request.

## Abbreviations

ATP: Adenosine triphosphate
EMT: Epithelial–mesenchymal transition
FAK: Focal adhesion kinase
JAK: Janus kinase
mtDNA: Mitochondrial DNA
NEDD4: Neural precursor cell-expressed, developmentally down-regulated protein 4
OXPHOS: Oxidative phosphorylation
POLG: DNA Polymerase gamma
PRRG4: Proline-rich gamma-carboxyglutamic acid protein 4
Src: V-src avian sarcoma (Schmidt-Ruppin A-2) viral oncogene homolog
STAT3: Signal transducer and activator of transcription 3
WAGR: Wilms tumor, Aniridia, Genitourinary anomalies, and mental Retardation
