# Supplementary material for: PRRG4 regulates mitochondrial function and promotes migratory behaviors of breast cancer cells through the Src-STAT3-POLG axis
Source: Cancer Cell Int. 2023 Dec 16;23:323. doi: 10.1186/s12935-023-03178-0 (PMC10724894; doi:10.1186/s12935-023-03178-0)
Supplement: Supplementary file 1 — Additional file 1: Table S1. Primer sequences for subcloning and point mutagenesis. Table S2. The sequences of the primers for q-PCR. [file 12935_2023_3178_MOESM1_ESM.docx]

Table S1 Primer sequences for subcloning and point mutagenesis.

| Primer name | Primer Sequence (5' to 3') |
| --- | --- |
| -1516/+155-POLG | F: GACGCGTCACTGTGGCTGACAGCCTATGCTCA |
|  | R: GAAGCTTAACCTCGGTCCTCACGGTGCTT |
| -994/+155-POLG | F: GACGCGTATCAGCTGTGTGATCTCGGTTGGTC |
|  | R: GAAGCTTAACCTCGGTCCTCACGGTGCTT |
| -1516/+155-POLG-Mut1 | F: GGCGGAGGATTGTTGCTCTGCACCCCACTCCTTAAA |
|  | R: GACTTTCGCGTTTTTAAGGAGTGGGGTGCAGAGCAACA |
| -1516/+155-POLG-Mut2 | F: ACATTGTGTATATTAAGTACATCTAAACAAGTGTATG |
|  | R: ATGAAATATTTTGCATACACTTGTTTAGATGTACTTAA |

Table S2 The sequences of the primers for q-PCR.

| Primer name | Primer Sequence (5' to 3') |
| --- | --- |
| *β*-actin | F: CGGTTCCGTTGCCCTGAGGCTCTT |
|  | R: CGTCACACTTCATGATGGAATTGA |
| MT-ND1 | F: CCCATGGCCAACCTCCTACTCCTC |
|  | R: AGCCCGTAGGGGCCTACAACG |
| MT-ND2 | F: AACCCTCGTTCCACAGAAGCT |
|  | R: GGATTATGGATGCGGTTGCT |
| MT-ND3 | F: AAAATCCACCCCTTACGAGTG |
|  | R: GTTTGTAGGGCTCATGGTAGG |
| MT-CYB | F: CCCACCCTCACACGATTCTTTA |
|  | R: TTGCTAGGGCTGCAATAATGAA |
| MT-CO1 | F: GCCATAACCCAATACCAAACG |
|  | R: TTGAGGTTGCGGTCTGTTAG |
| MT-CO2 | F: ACCAGGCGACCTGCGACTCCT |
|  | R: ACCCCCGGTCGTGTAGCGGT |
| MT-CO3 | F: CCTTTTACCACTCCAGCCTAG |
|  | R: CTCCTGATGCGAGTAATACGG |
| MT-ATP6 | F: TTATGAGCGGGCACAGTGATT |
|  | R: GAAGTGGGCTAGGGCATTTTT |
| MT-ATP8 | F: CCCCATACTCCTTACACTATTCC |
|  | R: CGTTCATTTTGGTTCTCAGGG |
| 18S rDNA | F: TAGAGGGACAAGTGGCGTTC |
|  | R: CGCTGAGCCAGTCAGTGT |
| mtDNA | F: CACCCAAGAACAGGGTTTGT |
|  | R: TGGCCATGGGTATGTTGTTAA |
